# Tuning Textural Properties by Changing the Morphology of SBA-15 Mesoporous Materials

**DOI:** 10.3390/ma17122827

**Published:** 2024-06-10

**Authors:** Francisco Emanuel da Silva, Eduardo Rigoti, Mariele Iara Soares de Mello, Sibele B. C. Pergher

**Affiliations:** Laboratorio de Peneiras Moleculares, Instituto de Química, Universidade Federal do Rio Grande do Norte, Av. Senador Salgado Filho, 3000. Bairro Lagoa Nova, Natal 59072-970, RN, Brazil; francisco.silva.111@ufrn.edu.br (F.E.d.S.); rigoti.eduardo@gmail.com (E.R.); mellomariele@gmail.com (M.I.S.d.M.)

**Keywords:** SBA-15, morphology, mesoporous, mesopores

## Abstract

Changing the morphology is an excellent option for altering the textural parameters of SBA-15 materials. This study provides a guide on how the properties of mesoporous structures behave according to their morphology and their contribution to thermal stability. The objective of this work was to synthesize different morphologies (spherical, hexagonal prisms, rice-like grains, rods, and fibers) of SBA-15 materials and evaluate the existing textural changes. The materials were synthesized by varying the temperature of the synthesis gel from 25 °C to 55 °C, with stirring at 300 or 500 rpm. The results of X-ray diffraction, Fourier transform infrared spectroscopy, N_2_ adsorption and desorption, and scanning electron microscopy were evaluated. Thermal stability tests were also conducted in an inert atmosphere. The materials were successfully synthesized, and it was observed that they all exhibited different characteristics, such as their ordering, interplanar distance, mesoporous parameter, specific surface area, micropore and mesopore volumes, external mesoporous area, and wall thickness. They also presented different thermal stabilities. The rice grain morphology had the highest specific surface area (908.8 cm^2^/g) and the best thermal stability, while the rod morphology had the best pore diameter (7.7 nm) and microporous volume (0.078 cm^3^/g).

## 1. Introduction

Mesoporous silica materials are attracting wide interest due to their prospects as catalytic supports, as materials for adsorption process, and for flow and transport devices [1]. Nanostructured mesoporous silica has been widely used since its synthesis in 1992 by Mobil Corporation^®^, which developed the first mesoporous material, known as MCM-41, exhibiting a hexagonal mesopore arrangement [2]. Following the discovery of this material, a similar one emerged, known as SBA-15, discovered at the University of California, Santa Barbara in the United States [3].

The mesoporous material SBA-15 features a 2D hexagonal organization with a space group of *p6mm*. Unlike MCM-41, this material exhibits microporosity in its structure, which grants it greater thermal stability, opening the way for various applications [4,5]. The morphology of SBA-15 can easily be changed according to its desired application, which has drawn the interest of numerous researchers. As a result, various morphologies have been observed, such as fibers, rods, plates, rice-like grains, and spheres, all of which can be employed in different catalytic processes [6].

Changes in the morphology of SBA-15 occur through the synthetic process, which is highly relevant, as it allows for modifications to be made in its structural properties [7]. Many studies have shown that various synthesis factors can affect the morphology of these materials. These factors include the temperature of the synthesis gel, the agitation rate, and the aging temperature of the material, as well as the use of non-ionic additives, which can direct the material’s formation [8]. For example, spherical particles were observed with the modification of the synthesis process using the soft-template method [9].

The formation process of these materials is divided into three stages [10]. The first stage is the cooperative self-assembly of inorganic/organic composites. In the second stage, a new crystal-like phase is formed, which is rich in aggregates of block copolymer/silica species. The final stage involves the separation of phases from this liquid crystal-like phase and the subsequent growth of liquid crystals, driven by the further condensation of silica species [11,12].

As seen in the morphology of rods, it is possible to obtain a compact structure and shorter or longer channels. These morphologies offer different material dispersions on their surface, as well as varying diffusion rates in the reaction medium [13]. Such changes cause modifications to their structure, such as in their channel accessibility, pore diameter, and porous volumes, which are crucial for their application [14]. A spherical morphology is important for drug delivery; these materials provide the optimal performance when used with a natural polysaccharide [15].

The morphology of ordered mesoporous materials can alter the selectivity in catalytic reactions. A comparison between fibrous morphology (commonly found in SBA-15 materials) and dendritic fibrous morphology illustrates that materials with altered morphology exhibit greater structural resistance in the reaction medium, resulting in different values for the final conversion of products [16]. A significant example is the conversion and selectivity during the hydrogenation of carbon dioxide. In a study, rod-like and plate-like morphologies were used and doped with zirconium (Zr), leading to different percentages of dispersion within the mesopores and, consequently, altering the acidic sites on the surface of each material [17].

The dispersion and selectivity are related to the textural parameters of the materials, such as the pore diameter, specific surface area, and channel size [18]. Larger pores are necessary for adsorption processes due to the high accessibility of molecules in their channels [19,20]. Materials with a higher specific surface area are particularly important for heterogeneous catalysis because they can significantly increase the activity of the catalysts incorporated into their mesoporous structure, thereby increasing the reaction surface area [21,22,23].

Many studies have addressed the use of different morphologies of SBA-15; however, they do not explain why one material is better than others. Because their application depends on their textural properties, such as the specific surface, porous diameter, and volume, it is necessary to know which property will be appropriate and which morphology will be obtained. This study shows the textural properties that can be obtained by modifying the morphology of SBA-15 materials, presenting a guide for their necessary applications.

The mesoporous SBA-15 material was synthesized using the hydrothermal method. To analyze the results, its X-ray diffraction (XRD) patterns were observed to investigate the mesopore ordering. Images of the morphology were obtained using scanning electron microscopy (SEM), and finally, textural analysis was carried out using N_2_ adsorption and desorption at 77 K. Stability tests were conducted in an inert atmosphere to evaluate the influence of the textural properties. These tests are essential for understanding how materials behave under different conditions and how their structural characteristics may change over time.

This comprehensive approach provides valuable insights into how different morphologies affect the textural properties of SBA-15 materials, aiding the understanding of their potential applications and optimizing their performance in various fields.

## 2. Materials and Methods

### 2.1. Materials

Tetraethyl orthosilicate P.A. (TEOS; purity of >98.0%), poly(propylene glycol)-block-poly(ethylene glycol)-block-(propylene glycol) (Pluronic^®^ P123; average Mw of ~5800), and hydrochloric acid P.A. (HCl; 37 wt%) were obtained from Sigma-Aldrich (St. Louis, MO, USA).

The synthesis of SBA-15 with the traditional fiber morphology was based on the study by Zhao et al. (1998) [4], where the synthesis gel had the following molar composition ratio: 1 TEOS/0.017 P123/5.9 HCl/194 H_2_O. The morphologies of the spheres, hexagonal tubes, rice grains, and rods followed the method detailed by Lee et al. (2010) [24], which employed the following molar composition ratio: 1 TEOS/0.017 P123/6.2 HCl/162 H_2_O.

### 2.2. Synthesis of SBA-15 Materials with Different Morphologies

In the synthetic process of the material with the fiber morphology, 16.3 g of Pluronic P123 (EO20PO70EO20) was added to a solution containing 519.2 g of deionized water and 96.7 g of HCl. The mixture was stirred for 3 h at 35 °C to dissolve the copolymer. After 3 h, 34.8 g of TEOS was added. This solution was further stirred for 20 h and then transferred to a 1 L capacity Teflon autoclave and placed in an oven at 100 °C for 48 h. Finally, it was filtered until equilibrium pH (pH = ±7) was reached.

For the synthesis of the materials with spherical, hexagonal prism, rice grain, and rod morphologies, solutions were prepared by dissolving approximately 23.4 g of Pluronic P123 in 606.8 g of deionized water and 146.4 g of HCl. Each solution was stirred at 300 rpm for the spherical, hexagonal, and rod morphologies; 500 rpm was used only for the rice grain morphology. Different temperatures were adopted according to the morphologies: 25 °C, 30 °C, 35 °C, 40 °C, and 55 °C, respectively. The solution was stirred for about 3 h to reach thermal equilibrium. Then, 50 g of tetraethyl orthosilicate (TEOS, 98%) was added without interrupting the stirring, and the mixing was continued for approximately 1 h. After this period of agitation, the solution was placed in a static oven without any temperature change for 24 h. Subsequently, it remained static at 100 °C for an additional 24 h. Finally, it was filtered until equilibrium pH (pH = ±7) was reached. All synthesis parameters are summarized in Table 1.

All samples underwent a calcination process to remove organic material used as a template in their synthesis route. The heating rate was set to 2 °C min^−1^ up to a temperature of 600 °C, and they were held at this temperature for 6 h.

### 2.3. Characterizations

The synthesized materials were characterized with X-ray Diffraction (XRD) using a Bruker D2Phaser equipped with a Lynxeyer detector (Stockholm, Sweden) and copper radiation (CuKα λ = 1.54 Å) with Ni filter, 10 mA current, voltage of 30 kV and range 2 (theta) between 0.5° and 5°. Other configurations included a 0.1 mm diverging slit, a 0.5 mm central slit, and a 3 mm converging slit, with a step size of 0.02° and an acquisition time of 0.3 s.

Through the interplanar distances (*d*) in the (100) plane for materials with a hexagonal structure, it was possible to determine the mesoporous parameters (*a*_0_). The calculation consisted of first determining the distance corresponding to the main plane, following Bragg’s Law, as described in Equation (1) [25]:

𝜆𝐶𝑢 *Kα* = 2𝑑_ℎ𝑘𝑙_ sin 𝜃 (1)

With the values of the interplanar distance determined, it was possible to obtain the values of the mesoporous parameters of each material. For the obtained hexagonal materials, Equation (2) was used [26]:

𝑎_0_ = 2𝑑_100_/3 ^½^ (2)

The textural properties of the synthesized materials were investigated using N_2_ adsorption and desorption, employing a Micromeritics ASAP 2020 instrument (Norcross, GA, USA). The method was based on the N_2_ physisorption on the material at a temperature of 77 K (N_2_ condensation temperature). The materials underwent a pre-treatment at 150 °C for 2 h under vacuum to remove physically adsorbed water from their surface.

Using the data obtained from the adsorption and desorption isotherms, the t-plot method was applied to determine the micropore volume and external surface area. The Gurvich rule was applied to obtain the mesopore volume, BET was used for the specific surface area, and BJH was used to determine the mesopore distribution.

The morphology images of the materials were obtained using a TESCAN MIRA 4 scanning electron microscope (Brno, Czech Republic), utilizing an in-beam secondary electron detector with an energy of 10 KeV. The samples were coated with a thin layer of gold using a Denton Vacuum Desk V model evaporator (Moorestown, NJ, USA) at a voltage of 30 for 60 s.

Fourier transform infrared spectroscopy (FTIR) was carried out to observe the vibrational bands present in the structures of all synthesized materials. The FTIR spectra were obtained using the Shimadzu IRAffinity1 Fourier transform infrared spectrophotometer (Kyoto, Japan) in the range of 4000 to 400 cm^−1^ and with a resolution of 4 cm^−1^.

Thermal stability tests were performed for all materials using the thermogravimetric analysis (TGA) technique. The data were obtained using the Netzsch TG 209F3 instrument (Waldkraiburg, Germany), utilizing an alumina sample holder. A heating ramp of 10 °C·min^−1^ up to 900 °C followed by cooling was conducted under a nitrogen atmosphere. Subsequently, all samples underwent analysis using low-angle X-ray diffraction.

## 3. Results

Table 1 summarizes the morphologies of each material synthesized, as well as the stirring time, static time, and textural properties. All samples were obtained with the same stirring time of 60 min; however, a speed of 500 rpm was used for the rice grain morphology and 300 rpm was used for the others. The temperature was modified between 24° and 55° for the same static time, 24 h, to obtain unique textural properties. Among the materials synthesized, SB-SPH had the smallest pore diameter; SB-HEX had the widest silica wall thickness, which is important for thermal stability; SB-FIB had the most continuous mesoporous channels; SB-RIC presented the largest surface area, which is important for different applications of adsorption; and SB-ROD had the largest pore diameter.

**Figure 1:**
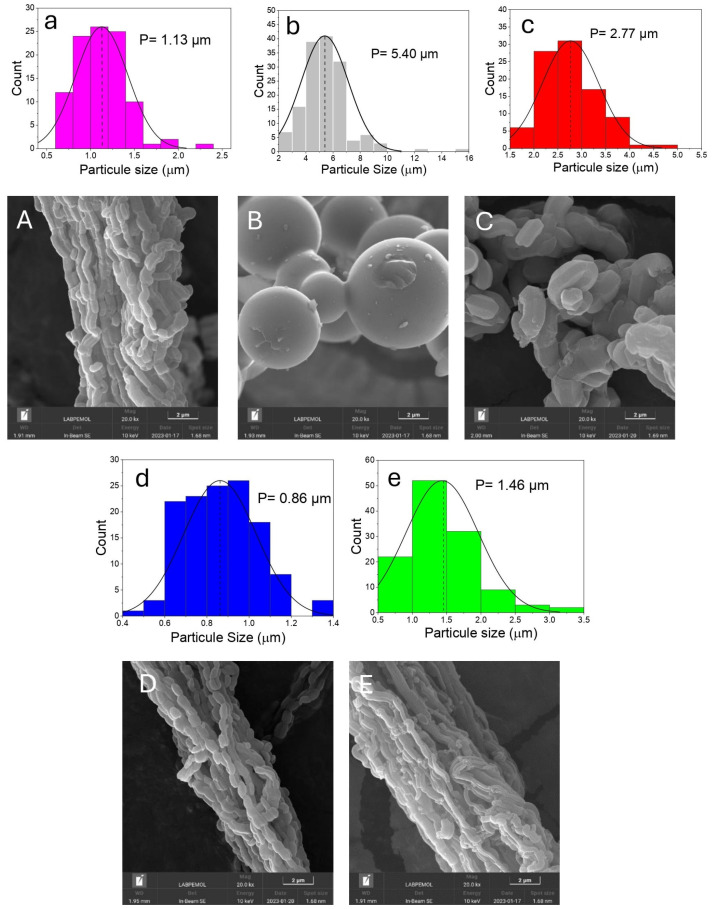
Particle size distribution (lower case) and Scanning electron microscopy-SEM (upper case) images of SB-FIB (**A**,**a**), SB-SPH (**B**,**b**), SB-HEX (**C**,**c**), SB-RIC (**D**,**d**), and SB-ROD (**E**,**e**).

Figure 1A,a shows the typical SEM images. It can be observed that the fibrous SBA-15 morphology (SB-FIB) was formed by an agglomeration of small particles with cylindrical shapes that were chemically connected at their ends, providing continuous mesoporous channels, a characteristic of this material. The average particle size of this material was around 1.13 µm. Figure 1B,b shows the successful formation of the spherical morphology, indicating that the synthesis temperature of this material favors this form during the primary particle growth step. This suggests that the value of ΔG in the medium led to the surface modeling the lowest possible surface tension [24]. However, this spherical morphology broke the continuity of the channels, leading to a loss of ordering. This morphology presented a particle size distribution with sizes around 5.39 µm.

Figure 1C,c presents SB-HEX, the material with hexagonal prism morphology. This material had well-defined particles and short channels that improved the diffusion within the pore structure. The synthesis temperature (30 °C) promoted a decrease in the particle size in comparison to that of the spherical material, 2.51 µm. Figure 1D,d presents the material with the rice grain morphology, which had the smallest particle size among all the materials. In the literature, it is highlighted that the combination of stirring and temperature leads to the rapid precipitation of silica, limiting particle growth in the nucleation stage [24].

The material obtained with rod-like morphology (Figure 1E,e) exhibited fine and elongated particles throughout its formation. This occurred due to the growth of more elongated primary particles, aiming to reduce the related free energy (ΔG) in the medium [27]. This morphology presented greater particle homogeneity, with an average particle size of 1.44 µm.

**Figure 2:**
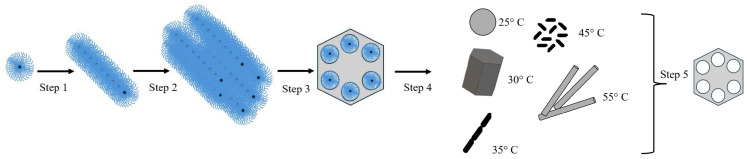
Resume of the synthesis mechanism of SBA-15 material with different morphologies: spherical (25 °C), hexagonal prism (30 °C), fiber (35 °C), rice grains (40 °C), and rod (55 °C).

Figure 2 shows the synthesis mechanism of the SBA-15 materials with different morphologies using different temperatures. In step 1, polymers dissolved in an acidic medium organized themselves into a micellar structure and formed organic tubes. This occurs in acid solution by hydrolysis process. In step 2, these tubes were grouped together to form a liquid crystal; here, the concentration is important to ordering. In step 3, the silicate ions dissolved in solution condensed into a formed structure of primary particles. In step 4, particle growth occurred, the silica polymerization was greatly influenced by synthesis parameters and the shape of primary particle, different temperatures led to different formations as the energy of the system was modified, and the morphologies reached equilibrium (see Scheme 1 in [28]). Finally, step 5 involved the removal of the matrix from the organic structure after the calcination process while maintaining hexagonal symmetry [2,28].

From steps 1 to 2, all materials have the same shape, however, in step 03 the temperature has a great influence. At this step, the formation of the primary particle occurs, followed by the particle’s growth. In step 4, the polymerization of silica can be rapid at the highest temperature (55 °C), where the weak van der Waals interaction controls it, preserves the shape of the previous step, and has smaller particles. This process is slow at the lower temperature (25 °C), where strong association and incomplete polymerization of silica may form large particles [29,30,31].

Temperature and agitation give to the system different free energy, and consequently the modification of morphologies. This occurs, because the particle grows searching the decrease surface tension [10]. So, the morphologie s change: at 25 °C, it has a spherical shape, and at 30 °C and, it has a form similar to a hexagonal prism. Both SB-SPH and SB-HEX have large sizes. When the temperature is increased, the materials display different shapes, such as fibrous (SB-FIB) at 35 °C, rice grain (SB-RIC) at 40 °C, and rod (SB-ROD) at 55 °C, and the particle is smaller.

**Figure 3:**
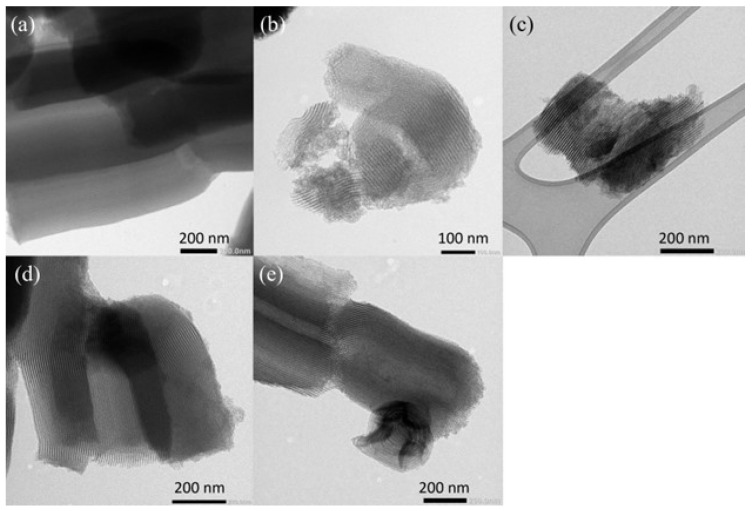
Transmission electron microscopy (TEM) images of SB-FIB (**a**), SB-SPH (**b**), SB-HEX (**c**), SB-RIC (**d**), and SB-ROD (**e**).

All TEM images of the morphologies are shown in Figure 3. In Figure 3a, showing SB-FIB, it can be seen that the mesoporous channel had higher orderliness and continuity. This has been observed in other reports in the literature on fibrous SBA-15. This is an important feature of this material because it directly influences its catalytic diffusion. SB-SPH, shown in Figure 3b, presented deformed mesoporous channels with different sizes. This happens because the morphology changes the channel directions, making the surface more accessible, but it can also obstruct its porosity. The SB-HEX (Figure 3c) material had short channels compared to the fibrous morphology because the particles broke the connections between the channels, improving the diffusion and maintaining the ordering. Figure 3d shows that SB-RIC had the smallest particle size and mesoporous channels, but it did not have any negative modifications in its ordered structure. The SB-ROD material (Figure 3e) had a loss of continuity in its channels because the connections were broken in its structure. However, this did not affect its order, so it is good for porous diffusion in catalyst applications.

**Figure 4:**
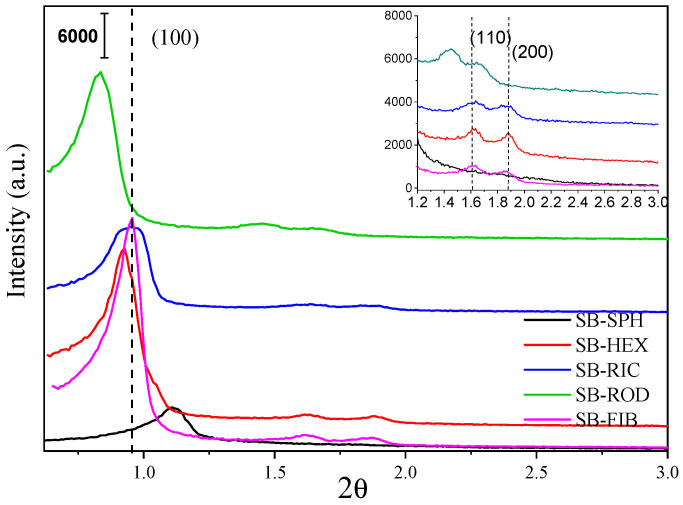
X-ray diffraction patterns of the SB-SPH, SB-HEX, SB-RIC, SB-ROD, and SB-FIB samples. The smaller graph shows figures of d_110_ and d_200_ in better resolution.

As shown in Figure 4, X-ray diffraction data were obtained for all samples to observe the ordering of the materials. All patterns show the peaks corresponding to the characteristic planes of the SBA-15 materials, with Miller indices of (100), which is the most intense peak of the material, and less intense peaks at higher 2θ values corresponding to (110) and (200) [32].

However, it can be observed that SB-SPH does not present a peak corresponding to planes at higher angles. This is because this material exhibited a decrease in its ordering, showing defects in its structure. Additionally, this material shows a shift in its (100) peak, indicating a modification in its mesopore parameter (a_0_) [33]. On the other hand, the SB-HEX, SB-RIC, and SB-FIB samples show peaks at the same positions for (110) and (200). However, in the hexagonal prism morphology, there was a slight shift in its main (100) peak, which was due to the modification in its mesoporous parameter, indicating an increase in its value.

The SB-ROD sample shows a significant displacement compared to the other samples, with shifts in all peaks of the material. This was due to a substantial increase in its mesoporous parameter, which modified the interplanar distance of the material, consequently shifting its position on the 2θ axis. Regarding the intensity, significant differences can be observed, indicating that all materials had different sizes of mesoporous channels. Materials with a higher diffraction intensity, such as SB-ROD, SB-HEX, and SB-FIB, had larger pore channels, with the fiber sample having the largest. Conversely, materials with a lower intensity show a reduction in their pore channels, as seen in the SB-RIC and SB-SPH samples.

The SB-FIB sample, with the commonly found fibrous morphology, exhibited an interplanar distance value of 9.24 nm (Table 2) for the (100) peak, with a 2θ position at 0.95°. This material had a mesoporous parameter (a_0_) of 10.70 nm. On the other hand, the SB-SPH material shows a slight decrease in its interplanar distance, measuring 9.22 nm, indicating the approximation of its planes due to its compact structure at the 2θ position of 1.11°. SB-SPH had the lowest mesoporous parameter value, making it the most compact material in terms of its pore system and material wall, with a value of 10.66 nm.

The opposite can be observed for the SB-ROD sample, with a d_100_ value of 9.82 nm, modifying the structure to make it more spacious, and causing the observed change in the X-ray diffraction pattern in Figure 3. Among all the samples, SB-ROD had the highest a_0_ value, making it the most spacious material in terms of its pore system and material wall, with a value of 11.70 nm. The same occurred for the SB-RIC sample, which shows a slight modification in the peak corresponding to (100) at 0.94° 2θ, increasing its interplanar distance value to 9.34 nm. This indicates a change in its a_0_ value, making this material less compact compared to the fibrous morphology material.

For the SB-HEX sample, an increase in its d100 is observed, measuring 9.61 nm, indicating a modification in its structure. This value is reflected in its mesoporous parameter, which was 11.09, making this material less compact than the SB-FIB material and causing a shift in the (100) peak, as shown in Figure 4.

**Figure 5:**
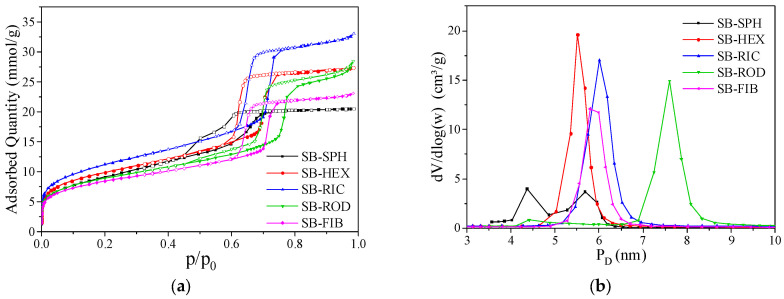
Adsorption and desorption isotherms and pore distribution: SB-SPH, SB-HEX, SB-RIC, SB-ROD, and SB-FIB.

In Figure 5a, nitrogen adsorption and desorption isotherms were obtained for all synthesized morphologies. All samples exhibited type IV isotherms, which are attributed to materials with cylindrical pores, indicating that adsorption occurred with the initial formation of monolayers on the mesopore walls until they were fully filled, forming the saturation plateau at higher relative pressures [34].

The ordered mesoporous materials exhibited type H1 hysteresis. This was observed in the materials that had homogeneity in their mesopore diameter, where the network effects were minimal, and the narrow and steep loop is a clear sign of delayed condensation. The SB-SPH material showed a peculiar hysteresis compared to the other synthesized materials. This occurred due to the loss of material ordering, as seen in the previously presented XRD, causing some blockage in the pores, trapping the gas, and resulting in its complete release only at lower partial pressures.

Regarding the amount adsorbed, it is evident that there were significant differences among the materials. The SB-RIC material had the highest absorption value, which was due to the reduction in its mesoporous channels, making them more accessible and causing complete filling. On the other hand, the SB-SPH material had smaller and less accessible pores due to its loss of ordering, making its pores less homogeneous. This led to distortion in the hysteresis and a lower intensity of the planes in the XRD.

The SB-HEX sample presented a higher adsorption value compared to the SB-FIB sample, a material commonly synthesized with a morphology featuring relatively smaller channels, similar to the SB-RIC material. This was due to the ease of filling its pores because its morphology caused the continuity of its channels to break.

The same behavior was also observed in the SB-ROD material. However, it is worth noting that at the end of the isotherm, at the near relative pressure (p/p^0^) of 1, there is a last point indicating a slightly higher adsorbed quantity than the samples with hexagonal prism and fiber morphologies. This was due to its larger pore diameter. When completely filling the channels, this material can adsorb a greater amount at higher pressures, until reaching its saturation plateau. Additionally, it can be observed that, due to the increased pore diameter, the monolayer filling and pore saturation occurred at higher pressures, shifting the isotherm to the right.

The average pore distribution was calculated for the samples with different morphologies (Figure 5b). The SB-SPH material exhibited two distinct pore diameters, which was a result of the loss of ordering. This indicates that the initial synthesis temperature affected the structure of these pores, with lower temperatures affecting smaller pore diameters [35]. On the other hand, the SB-HEX sample shows slightly smaller pores compared to SB-FIB. This was due to its synthesis temperature, which led to a reduction in the pore size, but with a more homogeneous distribution.

The SB-RIC sample had slightly larger pore diameters than the fiber morphology, as its synthetic process involved an increase in the synthesis temperature, while still demonstrating high homogeneity of its pores. This same behavior was observed in the SB-ROD sample, which had the largest pore diameter among all the samples, indicating that reaching higher temperatures led to changes in its pore size.

In Table 3, the values for the textural properties of all synthesized samples can be observed. Using BET calculations, the specific surface area (S_BET_) of SB-FIB was determined to be 687.9 m^2^/g. T-plot analysis was used to calculate the values of the mesoporous and microporous volumes, which were 0.732 cm^3^/g and 0.072 cm^3^/g, respectively. The external surface area value was also obtained, with a value of 504.6 m^2^/g. This value indicates the portion of the surface area that does not include the micropore area, meaning it represents the area attributed to the mesopores of the material. The wall thickness (W_0_) was calculated by subtracting the average pore diameter from the mesoporous parameter value, resulting in a value of 4.3 nm for this material.

For the SB-SPH material, with a spherical morphology, we observed a higher specific surface area (S_BET_) compared to the fiber morphology, with approximately 736.6 m^2^/g. There was also an increase in the external surface area (S_EXT_) of this material, with a value of 678.31 m^2^/g. This increase was due to a reduction in its microporosity, resulting in a higher specific area attributed to its mesopores.

Regarding the volumes of mesopores (V_meso_) and micropores (V_micro_), we obtained values of 0.92 cm^3^/g and 0.021 cm^3^/g, respectively. These values represent significant decreases compared to SB-FIB and the other materials. These reductions were due to the decreases in the pore diameters to 4.3 and 5.7 nm, causing N_2_ trapping and deforming the hysteresis behavior of the material. Regarding the wall thickness (W_0_), there was an increase compared to SB-FIB, to approximately 5.0 nm. This increase was a result of the reduction in the pore diameter.

Thus, increasing the volume of N_2_ adsorbed in the isotherm was due to the pore breaking provided by the morphology, making the channels more accessible and, consequently, increasing the mesoporous volume. The pore diameter slightly decreased to 5.6 nm, indicating that the major contribution to the modifications in the porous volumes of the material was the change in the morphology and its effects on the channel system. The wall thickness increased to 5.5 nm, consequently causing an increase in the mesoporous parameter. The synthesis temperature of 30 °C led to the formation of a thicker wall without drastically reducing the pore diameter, resulting in a less compact hexagonal structure.

The SB-RIC material exhibited a significant increase in its specific surface area (S_BET_), reaching 908.8 m^2^/g. This increase in the surface area is attributed to the increase in its external surface area (S_EXT_), which also rose to a value of 724.96 m^2^/g. This expansion was a result of the mesoporous volume, which reached 1.075 cm^3^/g. The increase in mesoporous volume was due to the shortening of the channels, facilitating accessibility and leading to a more complete adsorption of N_2_, resulting in a higher volume of adsorbed gas, as shown in the isotherm.

The microporous volume of this material remained the same as that of SB-FIB and increased compared to SB-SPH and SB-HEX, with a value of 0.072 cm^3^/g. This microporous volume is crucial for the material’s thermal stability.

Regarding the pore diameter, there was an increase to 6.1 nm, causing a slight shift in the main peak observed in the X-ray diffraction analysis. This factor also enhances its adsorption capacity for obtaining the isotherm, contributing to the increase in the external surface area due to the increase in accessibility to the mesoporous channels. Additionally, there was an increase in the wall thickness, consequently increasing the mesoporous parameter.

Regarding the SB-ROD material, it obtained a specific surface area of 724.8 m^2^/g and an external surface area of 532.11 m^2^/g. This represents an increase compared to the SB-FIB and SB-SPH samples and a decrease compared to the SB-HEX and SB-RIC samples. The increase in the specific surface area is attributed to the larger mesopore diameter, reaching 7.6 nm. This material was synthesized at 55 °C, and with the increase in the synthesis gel temperature, there was an expansion of the mesopore diameter. Due to the increased mesopore diameter, the wall thickness (W_0_) of the material decreased to a value of 3.7 nm.

**Figure 6:**
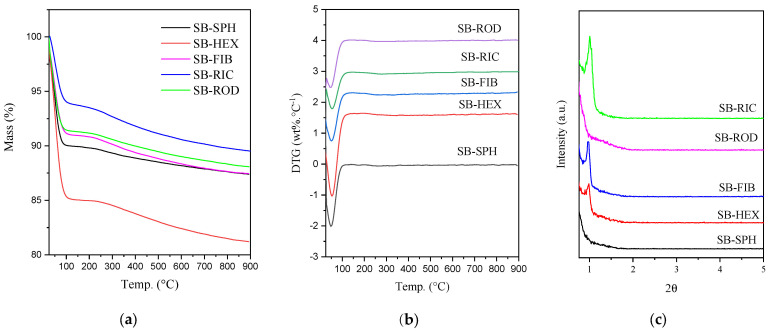
(**a**) Thermogravimetry (TG), and (**b**) derivative thermogravimetry (DTG), and X-ray diffraction after thermogravimetrial analysis (**c**).

The sample materials showed only a single mass loss, as seen in Figure 6a. This loss occurred at temperatures near 100 °C, indicating that it corresponds to the release of water present in the material’s structure. SB-HEX contained a larger amount of water in its structure, as it exhibited a higher mass loss. The presence of a single event was confirmed using the DTG method, as shown in Figure 6b, which indicates the material loss during the evaporation of water molecules from the surface.

X-ray diffraction patterns were obtained after the thermal analysis of the materials. It can be observed that some samples exhibited different intensities, with the material with rice grain morphology showing better stability and better resolution of its main peak. This is followed by the material with hexagonal prism morphology, which maintained excellent ordering, an improvement compared to the material with fiber morphology, commonly synthesized in the literature, which showed lower structural stability.

For the samples with spherical and rod morphologies, a total loss of ordering can be observed, indicating that these materials lost their thermal stability at high temperatures. This loss of ordering in the spherical material led to instability in the channel structure. Regarding the rod-shaped material, there was a reduction in its mesoporous wall, making its structure more sensitive to temperature increases.

The superficial area (S_BET_) and the porous system (ordering and size) are very important for thermal stability. When the material is subjected to higher temperatures, the oxygen in Si-OH can be removed (as H_2_O) to make Si-O-Si, closing microporous and reducing mesopores diameter, and condensing the structure [36,37]. Thus, the materials that have low wall thickness and low specific surface area can collapse. Pore diameter with heterogeneous sizes have bigger impact in the stability, the structure condensation is very sensitive. Thus, the material SB-RIC shows higher ordering after thermal analysis (highly stable), while SB-SPH and SB-ROD do not present characteristic peaks 001 in XRD (less stable materials).

## 4. Discussion

The textural properties of mesoporous materials are always being improved, given the importance of expanding their applicability. Modifications through changes in morphology are effective and crucial for obtaining accessible and adjustable materials. The results of our study show that when we lowered the synthesis temperature to obtain a spherical morphology, this material exhibited a reduction in its mesoporous ordering, which has been reported in various studies in the literature [38,39].

The improvement in the thermal stability exhibited by the rice grain morphology is highly intriguing for processes requiring high temperatures, as it is well-documented in the literature that these materials undergo significant structural loss at elevated temperatures [40,41]. This is particularly relevant for the morphology of fibers, which are commonly synthesized. This presents an opportunity for industrial-scale applications, a property that can be further enhanced through the incorporation of metals [42,43].

A relationship between the intensity of the peaks present in the X-ray diffraction (XRD) pattern of each sample and their channel systems was observed, where the SB-RIC material exhibited a decrease in its peaks, indicating a reduction in its channels, which was confirmed by the N_2_ isotherm, similar to what was observed by Gao et al. [43]. This resulted in a higher amount of adsorbed material. Conversely, the decrease in the peaks present in SB-SPH was associated with the loss of its ordering and a reduction in its mesopores, which affected its N_2_ isotherm, resulting in lower adsorption and deformation in its hysteresis.

Furthermore, the intensity and peak shifts observed in the XRD pattern of the SB-ROD sample indicate the presence of longer channels and a possible larger pore diameter. Indeed, this material exhibited the largest pore diameter among all the samples, which enhanced its adsorption capacity performance. This observation aligns with the findings by Dacquin et al. [44], although their study involved the use of an acid chemical treatment, which may have led to material degradation.

The SB-HEX material exhibited a lower intensity, which is attributed to the breaking of its channels, a phenomenon confirmed by its isotherm. This also confirms that the morphological change provided better accessibility for the material, as there was a slight increase in the adsorbed amount compared to the SB-FIB material. It is essential to highlight that despite the different synthesis methods, the FTIR spectra of the materials (Figure S1, Supplementary Materials) showed bands corresponding to their structure, the bonding of their silanol groups Si-OH (954 cm^−1^), and the Si-O-Si (1046, 798 and 431 cm^−1^) bonds present in the material formation. This indicates that even with the attainment of different morphologies, the chemical characteristics of the structure remain unchanged [45].

The SB-RIC material exhibited the highest specific surface area among all the materials, and this is because this material had the smallest particle size among them, reducing its channels and, consequently, increasing its surface area. This is a crucial factor for its application in catalysis, for example. For the same reason, this material also showed the highest mesoporous volume, which in turn increased its external surface area, referring to the area of mesopores. This result is significant for various applications, highlighting the material’s enhanced textural properties and its potential for catalytic processes. This factor is crucial for applications in the fields of catalysis and adsorption, since this characteristic provides the material with a larger contact surface with the desired species, leading to better dispersion of active sites.

Among the SBA-15 materials, SB-ROD exhibited the highest microporous volume, which contradicts the findings in the literature, where it has been commonly observed that increasing the synthesis temperature decreases the microporous volume of this material. However, this synthesis method goes against that trend. As a result, its mesoporous area decreased, but this material presented the largest pore diameter among all the materials, directly impacting its mesoporous parameter, which turned out to be the largest among the materials with a hexagonal structure, making it the material with the most spacious structure.

Regarding the wall thickness, the SB-HEX material exhibited the highest value, which was caused by a slight reduction in its pore diameter, resulting in a slightly larger mesoporous parameter than expected for SBA-15 materials. This factor renders this material highly stable in processes that require high temperatures, providing an additional level of stability.

Changes in the textural properties based on the morphology prompt thoughts of future directions for this material, such as its diverse applicability in areas such as catalysis and adsorption, where different pore diameters and a high specific surface area are required to enhance activity due to increased contact. The improved stability of this material may lead to its consideration for applications in industries that require inert supports with high-temperature stability.

## 5. Conclusions

SBA-15 materials with different morphologies were obtained to observe their textural modifications. It was possible to enhance different factors, such as the specific surface area, microporous and mesoporous volume, and the external surface area. Moreover, modifications in the pore diameter and wall thickness were highly adjustable based on these morphological changes.

The size of the mesoporous channels can be adjusted differently in each morphology, based on the materials with distinct characteristics. SB-RIC material has garnered significant attention due to its increased textural factors and reduced channel size, making it a promising material for further study. Additionally, SB-ROD material has shown great potential for applications due to its larger pore diameters.

## Figures and Tables

**Table 1:** Summary of morphologies, synthesis parameters, and textural properties.

Materials	Morphology	Temperature (°C)	Speed (rpm)	Stirring Time (h)	Static Time (h)	Textural Properties
SB-SPH	Spherical	25	300	60	24	Smaller pore diameter
SB-HEX	Hexagonal prism	30	300	60	24	High silica wall thickness
SB-FIB	Fiber	35	300	60	24	Continuous mesoporous channels
SB-RIC	Rice grain	40	500	60	24	High surface area
SB-ROD	Rod	55	300	60	24	Larger pore diameter

**Table 2:** Values of d100 and a0 for SB-SPH, SB-HEX, SB-RIC, SB-ROD, and SB-FIB.

SBA-15	d_100_ (nm)	a_0_ (nm)
SB-SPH	9.2	10.7
SB-HEX	9.6	11.1
SB-RIC	9.3	10.8
SB-ROD	9.8	11.3
SB-FIB	9.2	10.7

d_100_ = interplanar distance; a_0_ = value for the mesoporous parameter.

**Table 3:** Textural analysis of SB-SPH, SB-HEX, SB-RIC, and SB-ROD materials.

Morphology	S_BET_ (m^2^/g)	V_meso_ (cm^3^/g)	V_micro_ (cm^3^/g)	S_ext_ (m^2^/g)	D_BJH_	a_0_	W_t_
SB-SPH	736.6	0.692	0.021	678.31	4.3–5.7	10.7	5.0
SB-HEX	774.3	0.882	0.068	630.51	5.6	11.09	5.5
SB-RIC	908.8	1.075	0.072	724.96	6.1	10.8	4.8
SB-ROD	724.8	0.909	0.078	532.11	7.6	11.3	3.7
SB-FIB	687.9	0.732	0.072	504.60	5.8	10.7	4.3

S_BET_ = surface area using the BET method; V_meso_ = volume of primary mesopores; V_micro_= volume of micropores; a_0_ = value for the mesoporous parameter; S_ext_ = external surface area using the t-Plot method; DBJH = pore diameter; W_t_ = silica wall thickness (W_t_ = a_0_ − D_BJH_).

## Data Availability

Data are contained within the article.

## Supplementary Materials

The following supporting information can be downloaded at: https://www.mdpi.com/article/10.3390/ma17122827/s1, Figure S1: Fourier Transform Infrared (FTIR) Spectrum for SB-FIB, SB-SPH, SB-HEX, SB-RIC, SB-ROD; Figure S2: Transmission electronic microscopy to SB-SPH; Figure S3: Transmission electronic microscopy to SB-HEX; Figure S4: Transmission electronic microscopy to SB-FIB; Figure S5: Transmission electronic microscopy to SB-RIC; Figure S6: Transmission electronic microscopy to SB-ROD.
