# Supplementary material for: Tuning Textural Properties by Changing the Morphology of SBA-15 Mesoporous Materials
Source: Materials (Basel). 2024 Jun 10;17(12):2827. doi: 10.3390/ma17122827 (PMC11204648; doi:10.3390/ma17122827)

**Figure S1.** Fourier Transform Infrared (FTIR) Spectrum for SB-FIB, SB-SPH, SB-HEX, SB-RIC, SB-ROD.

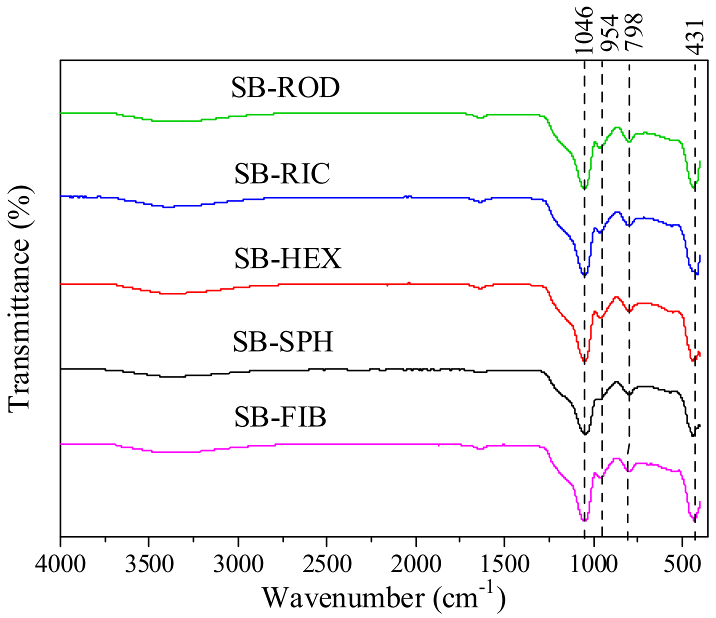

**Figure S2.** Transmission electronic microscopy to SB-SPH

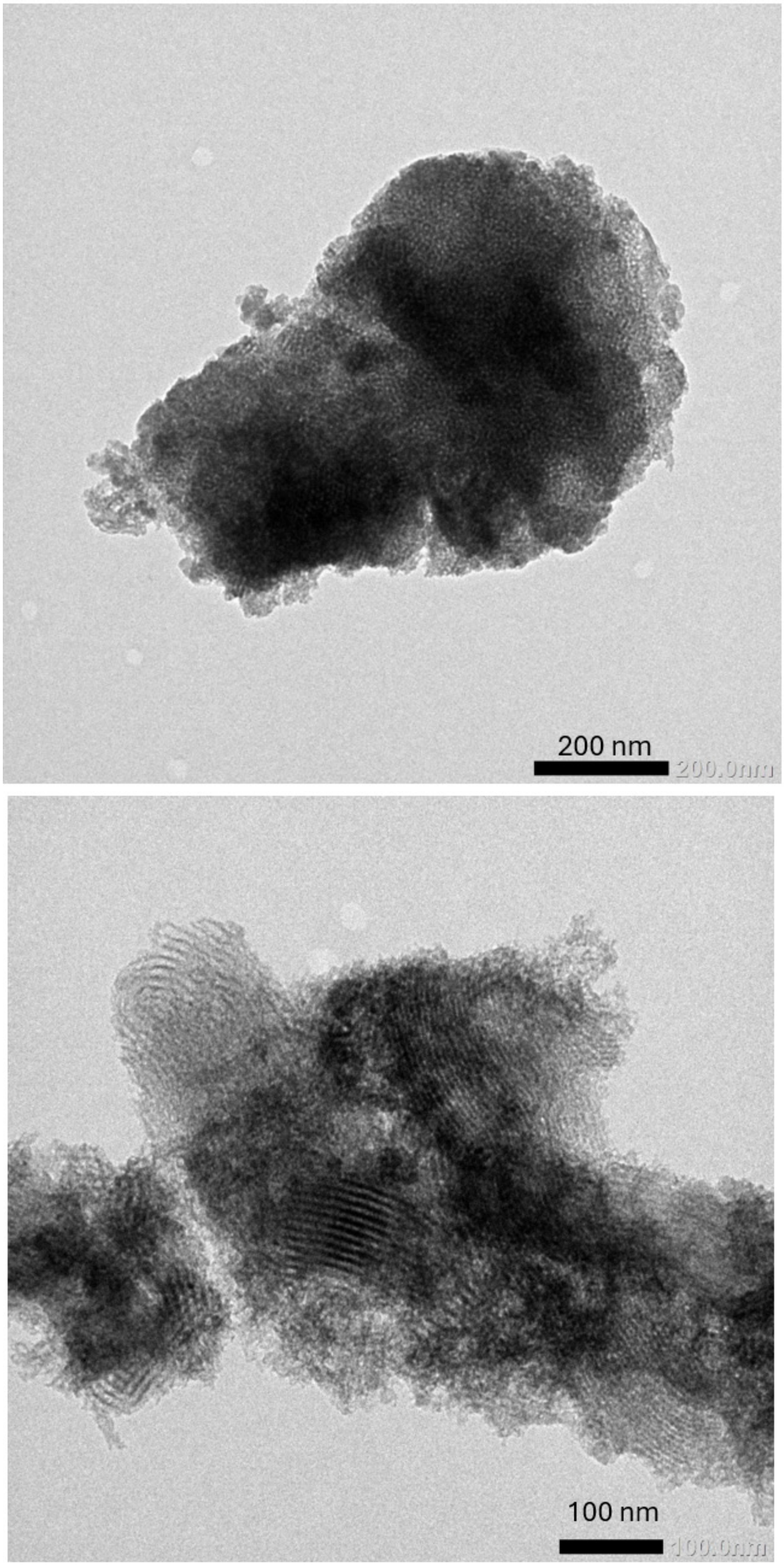

**Figure S3. -** Transmission electronic microscopy to SB-HEX

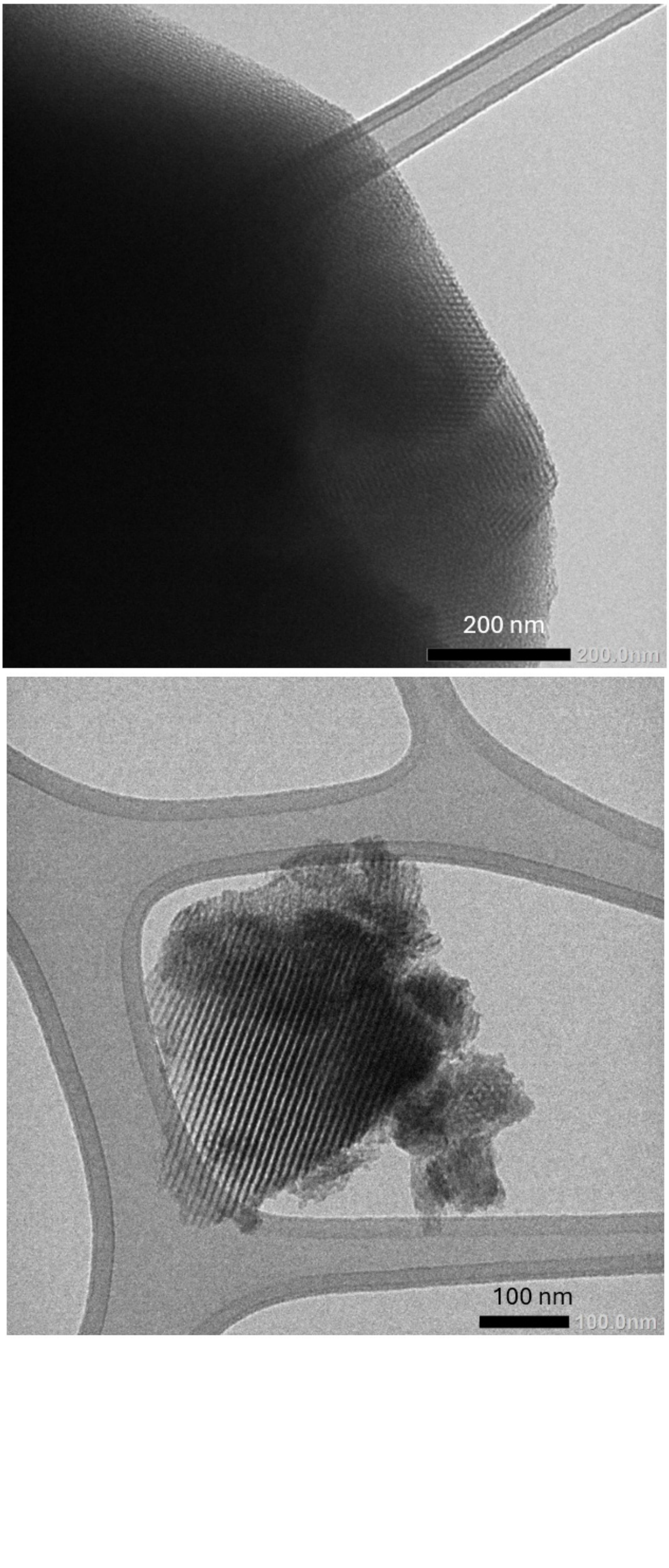

**Figure S4. -** Transmission electronic microscopy to SB-FIB

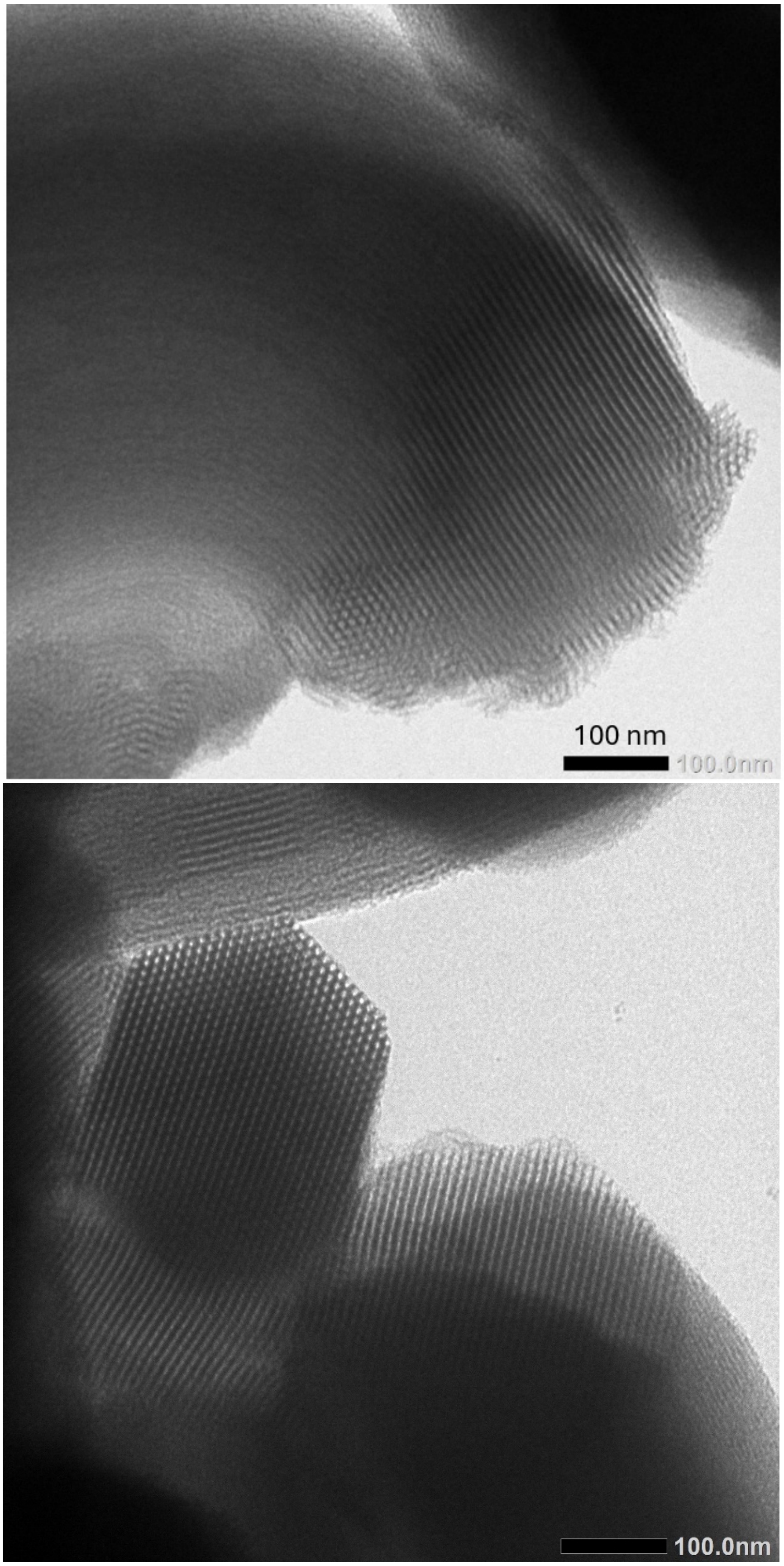

**Figure S5. -** Transmission electronic microscopy to SB-RIC

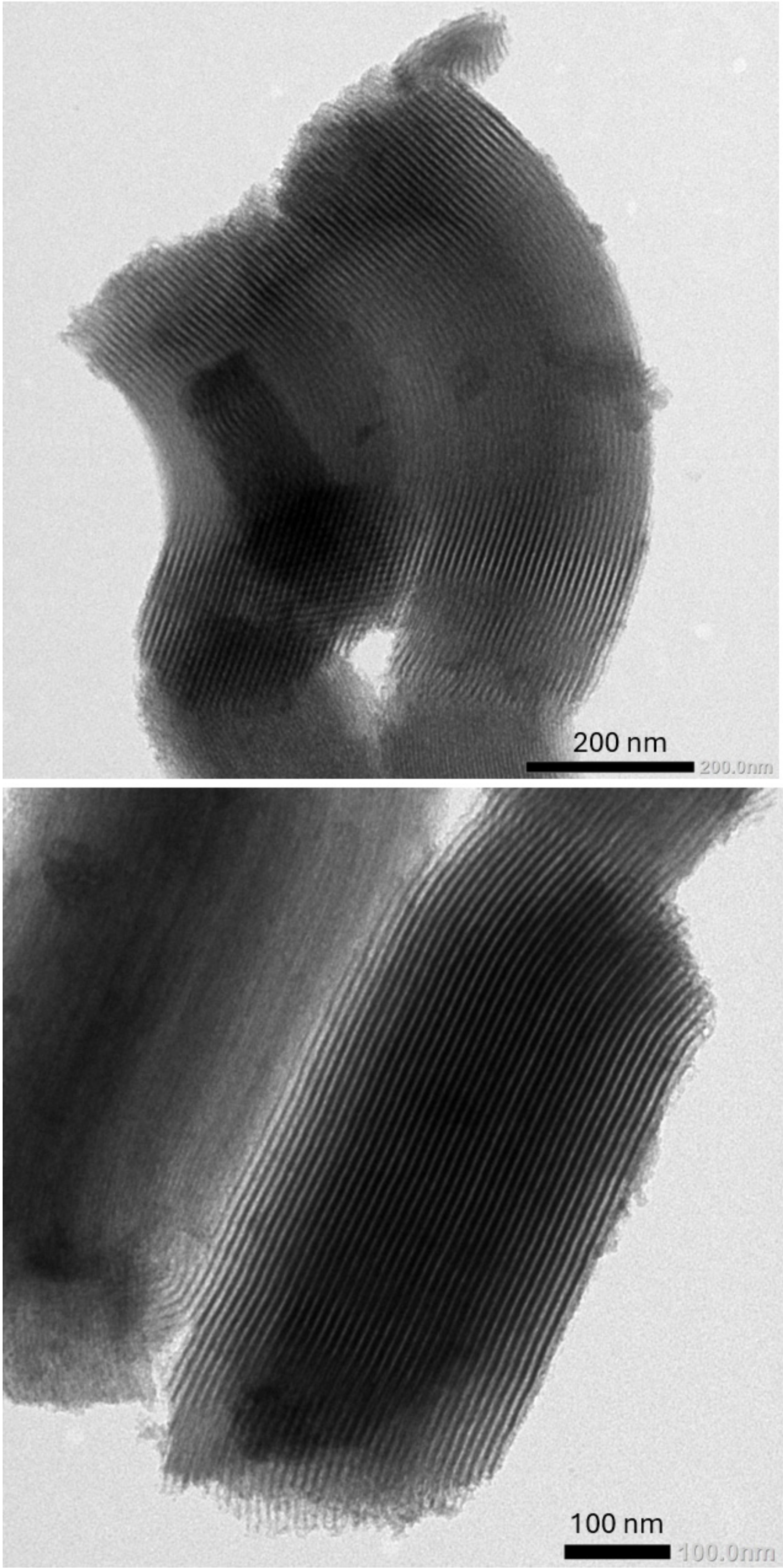

**Figure S6. -** Transmission electronic microscopy to SB-ROD

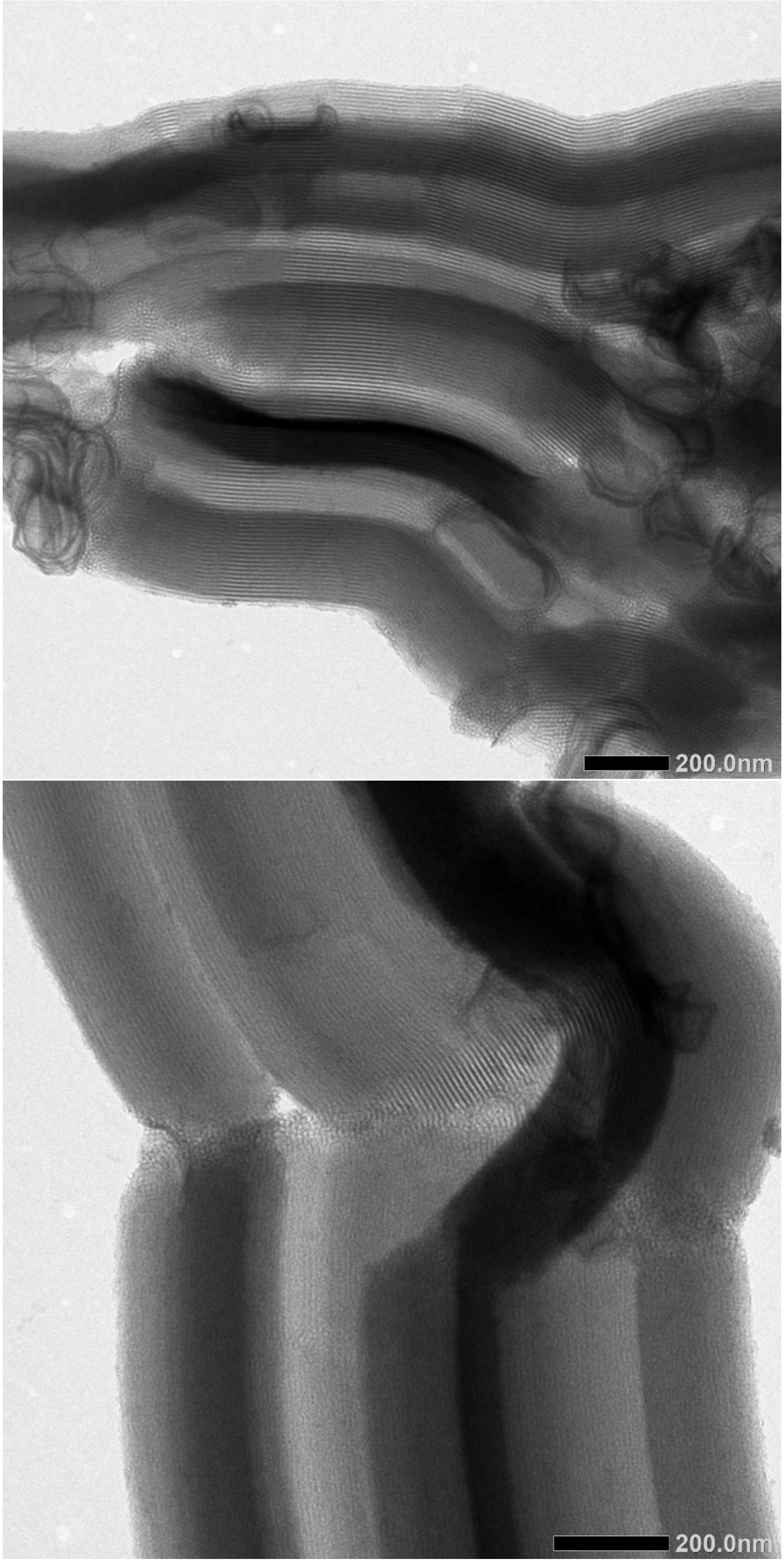

Supplement: Supplementary file 1 [file materials-17-02827-s001.zip › materials-2959588-supplementary.pdf]
